# Change of the cross-sectional area of vastus medialis oblique in patients with recurrent patellar dislocation treated by tibial tubercle transfer combined with medial patellofemoral ligament reconstruction on axial CT

**DOI:** 10.1186/s13018-022-03367-z

**Published:** 2022-10-28

**Authors:** Chao Zhao, Conglei Dong, Xiaomeng Wang, Lingce Kong, Bo Chang, Fei Wang

**Affiliations:** Department of Orthopaedic Surgery, Third Hospital of Hebei Medical University, No. 139 Ziqiang Road, Shijiazhuang, 050051 Hebei China

**Keywords:** Recurrent patellar dislocation, Cross-sectional area, Vastus medialis oblique, Tibial tubercle transfer, Medial patellofemoral ligament reconstruction

## Abstract

**Purpose:**

To investigate the change of the cross-sectional area (CSA) of vastus medialis oblique (VMO) in patients with recurrent patellar dislocation (RPD) treated by tibial tubercle transfer combined with medial patellofemoral ligament (MPFL) reconstruction by imaging methods, and to guide clinical treatment and rehabilitation.

**Methods:**

From October 2015 to March 2022, 23 patients with RPD who underwent tibial tubercle transfer combined with MPFL reconstruction were retrospectively enrolled. All patients were assessed by CT in the supine position with the knee fully extended and the quadriceps relaxed. The CSA of VMO and the ratio of CSA of VMO to body weight (CSA/BW) were measured at the upper pole of the patella, 5 mm above the upper pole of the patella and 5 mm below the upper pole of patella. The differences of measured parameters were compared before surgery and at follow-up, including CSA of VMO and CSA/BW. Test level *α* = 0.05.

**Results:**

The tibial tubercle-trochlear groove (TT-TG) distance was significantly reduced at follow-up compared with that before surgery (27.91 ± 1.95 mm vs 12.33 ± 1.07 mm, *P* < 0.001). The CSA of VMO was significantly increased at follow-up compared with that before surgery at 5 mm below the upper pole of the patella (473.06 ± 106.32 mm^2^ vs 562.97 ± 157.90 mm^2^, *P* < 0.001), at the upper pole of the patella (641.23 ± 188.45 mm^2^ vs 700.23 ± 177.55 mm^2^, *P* = 0.029), and at 5 mm above the upper pole of the patella (788.25 ± 238.62 mm^2^ vs 849.79 ± 180.84 mm^2^, *P* = 0.018). The CSA/BW was significantly increased at follow-up compared with that before surgery at 5 mm below the upper pole of the patella (7.83 ± 2.52 mm^2^/kg vs 9.22 ± 3.54 mm^2^/kg, *P* < 0.001), at the upper pole of the patella (10.48 ± 3.62 mm^2^/kg vs 11.42 ± 4.14 mm^2^/kg, *P* = 0.020), and at 5 mm above the upper pole of the patella (12.86 ± 4.65 mm^2^/kg vs 13.68 ± 3.86 mm^2^/kg, *P* = 0.017).

**Conclusion:**

After tibial tubercle transfer combined with MPFL reconstruction, CSA of VMO increased in patients with RPD, which will help to enhance patellar stability and reduce recurrence.

## Introduction

Recurrent patellar dislocation (RPD) is a common motor disease in young people with complex etiology [1, 2]. In anatomy, the occurrence of patellar dislocation is closely related to femoral trochlear dysplasia, patella alta, injury of the medial patellofemoral ligament (MPFL), lateralization of the tibial tubercle relative to femoral trochlear, abnormal torsion of the femur and tibia, and atrophy of vastus medialis oblique (VMO) [2–4]. 92% of MPFLs were damaged after the first acute patellar dislocation [5]. And MPFL reconstruction has become one of the basic operations for patellofemoral instability [6]. Further study confirmed that some patients with patellar dislocation had more lateralization of the tibial tubercle relative to femoral trochlear [7–10]. Tibial tubercle-trochlear groove (TT-TG) distance is one of the indicators used to measure the lateralization of tibial tubercle, which is important for surgical planning [11, 12]. MPFL reconstruction combined with tibial tubercle transfer improved functional scores in RPD patients with large TT-TG distance, compared with isolated MPFL reconstruction [13].

The vastus medialis is a component of the quadriceps femoris [14, 15]. The muscle fibers of the vastus medialis can be divided into two parts, especially the distal oblique part, which plays an important role in maintaining patellar stability during active knee extension and is known as VMO [16–19]. One study reported that the lateral stress of the patellofemoral joint increased by approximately 10% when the strength of the VMO was reduced [20]. Another study showed that improving the function of VMO reduced the pressure applied to the lateral patellofemoral articular cartilage [21]. Therefore, the recovery of VMO function will be very important for the prognosis of patients with patellar dislocation.

This study hypothesized that the CSA of VMO increased in patients with RPD after tibial tubercle transfer combined with MPFL reconstruction.

## Materials and methods

### Patients

From October 2015 to March 2022, 23 patients with RPD who underwent tibial tubercle transfer combined with MPFL reconstruction at the Third Hospital of Hebei Medical University were retrospectively enrolled. The present study was approved by the Ethics Committee of the Third Hospital of Hebei Medical University and all patients provided their informed consent for participation and publication.

Inclusion criteria were given as follows:

1. RPD (≥ 2 times);
2. Epiphysis closed;
3. TT-TG distance > 25 mm;
4. MPFL injury was observed by imaging data and during surgery;
5. Underwent tibial tubercle transfer combined with MPFL reconstruction;
6. Had relevant CT imaging data;
7. Follow-up time was not less than 1 year;

Exclusion criteria were given as follows:

1. Previous knee surgery;
2. Other knee joint diseases, such as fracture, ligament injury, and meniscus injury;
3. Patellofemoral arthritis or cartilage damage;
4. Severe knee valgus or varus deformity (> 5°) [22, 23];
5. Femoral or tibial torsion deformity [24, 25];
6. High grade femoral trochlea dysplasia (type B, C, D of Dejour’s classification) [26];
7. No MPFL injury was observed in imaging data or during surgery;
8. Other diseases or injuries affecting VMO, in addition to RPD;
9. Follow-up time less than 1 year or lost to follow-up;
10. Children or the elderly;

### Data collection and inspection

Patient information was collected, including age, sex, height, body weight (BW) before surgery and at follow-up, and body mass index (BMI) was calculated. Detailed radiological assessments of the lower limbs were performed before surgery and at follow-up. All patients were assessed by CT in the supine position with the knee fully extended and the quadriceps relaxed. The limbs were secured by devices to minimize movement. All examinations were performed using the same CT scanner.

### Surgical intervention

All patients were treated with tibial tubercle transfer combined with MPFL reconstruction.

All patients' surgeries were performed by the same team of senior surgeons. The patients were placed in supine position under general anesthesia. Tourniquet was tied around the base of the thigh. Arthroscopy was performed to evaluate the patellofemoral joint injury. A 4 cm incision was made along the lateral edge of the tibial tubercle to expose the tibial tubercle and its distal side. Tibial tubercle osteotomy was performed, and the bone block was appropriately displaced so that the TT-TG distance was 10-15 mm and Caton–Deschamps index (CDI) ≤ 1.1. The degree of tibial tubercle transfer was determined according to preoperative measurement parameters to avoid overcorrection. The displaced tibial tubercle was fixed with 2 screws in the optimal position. Under intraoperative C-arm fluoroscopy, the head end of the screws were threaded through the cortex to confirm the correct location of the tibial tubercle and screws.

The pes anserinus was located from the surgical incision of the original tibial tubercle, and the gracilis tendon was removed with a tendon extractor. Double-bundled patellofemoral ligament reconstruction was performed in all patients. The midpoint of the line between the medial epicondyle of the femur and the adductor tubercle served as the femoral stop of the graft. The inner upper horn and midpoint of the patella served as patella stops for the graft. The MPFL femoral insertion was determined by intraoperative fluoroscopy using the Schoettle point. The fixation method on the patellar side was to use 2 suture anchors. The femoral side was fixed with an absorbable screw.

After surgery, pressure dressing was performed for 48 h and bracing was performed for 6 weeks. Patients were allowed to use crutches for partial weight bearing during the 6 weeks. On the second day after surgery, rehabilitation training was started under the guidance of professional rehabilitation physicians, and normal activities were resumed within 6 months.

### Evaluation of results

**Fig. 1:**
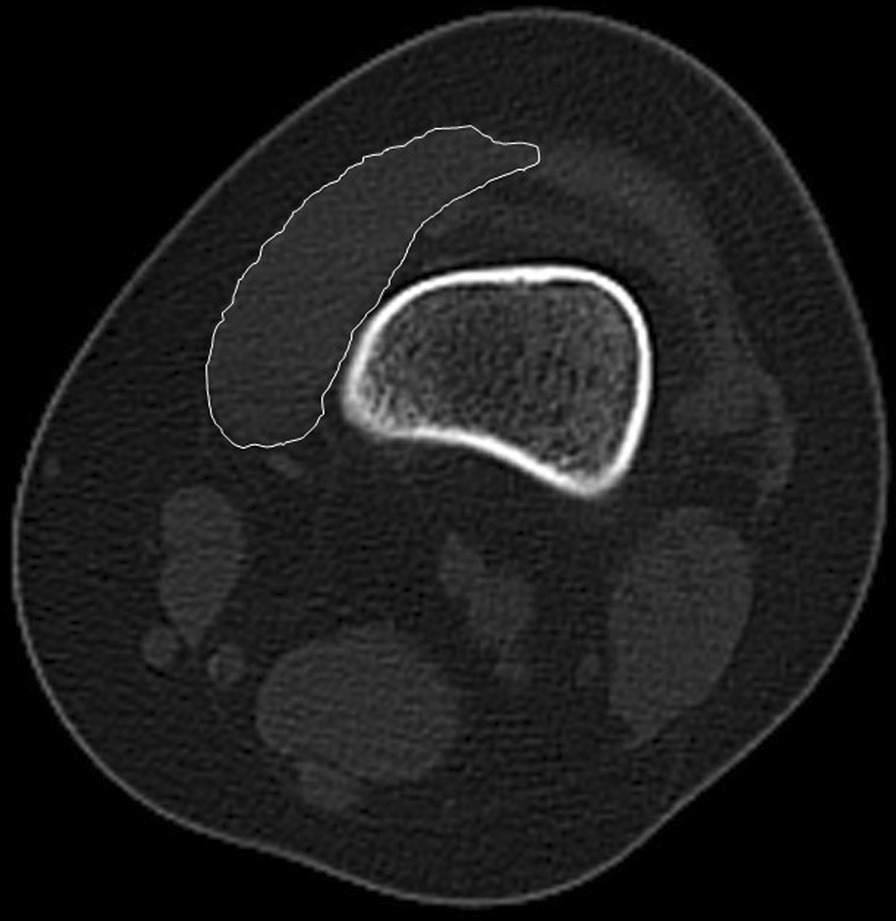
The cross-sectional area of vastus medialis oblique

CT images of knee joints were obtained for all patients by picture archiving and communication system (PACS). The CSA of VMO was measured using RadiAnt DICOM Viewer software (Medixant Ltd., Poznań, Poland). First, we made sure that the scans of the knee were in the same position. We measured the CSA of VMO at the upper pole of the patella, 5 mm above the upper pole of the patella and 5 mm below the upper pole of the patella. Referring to previous studies, the vastus medialis muscle in this section should have a high proportion of VMO [27–29]. The CSA was measured by manually delineating muscle boundaries by two trained observers (Fig. 1). The two observers were unaware of the patient's characteristics and obtained all measurements independently. Based on previous studies, the use of the ratio of CSA of VMO to BW (CSA/BW) was used to mitigate the effect of body weight on results [30].

### Statistics and analysis

SPSS21.0 software (Inc., Chicago, IL, USA) was used for statistical and analysis. A power analysis was undertaken. At least 21 cases were needed to reach an effect size of 0.65, a power of 0.80, and an alpha of 0.05 using G*Power v. 3.1 (G* Power, Dusseldorf, Germany). The Shapiro–Wilk test was selected for the normality test of continuous variables. Intraclass correlation coefficients (ICC) were calculated to test intra-observer and inter-observer reliability. The measurement data were described by mean and standard deviation, and evaluated by paired *T* test. The differences of measured parameters were compared before surgery and at follow-up, including CSA of VMO and CSA/BW. Test level *α* = 0.05.

## Results

### Demographic characteristics

In this study, 23 knees of 23 subjects were included with an average follow-up time of 1.39 ± 0.49 years, including 5 males and 18 females. Before surgery, the femoral anteversion angle was 15.83 ± 1.40°, the tibial torsion angle was 20.96 ± 4.53°, and there was no severe knee valgus or varus deformity (> 5°). According to Dejour’s classification, 6 patients had normal trochlear and 17 were type A. There were no significant differences in height, BW and BMI between the patients before surgery and the patients at follow-up. (Height: 164.52 ± 7.73 cm vs. 165.22 ± 6.78 cm, *P* = 0.158; BW: 63.70 ± 14.89 kg vs. 64.52 ± 14.95 kg, *P* = 0.081; BMI: 23.46 ± 5.06 kg/m^2^ vs. 23.57 ± 5.11 kg/m^2^, *P* = 0.510) (Table 1). No recurrence of patellar dislocation or complications were observed at follow-up.

**Table 1:** Demographic characteristics

Parameters	Before surgery	At follow-up	*P* value
Height	164.52 ± 7.73 cm	165.22 ± 6.78 cm	0.158
Body weight	63.70 ± 14.89 kg	64.52 ± 14.95 kg	0.081
Body mass index	23.46 ± 5.06 kg/m^2^	23.57 ± 5.11 kg/m^2^	0.510

### Measured parameters

Imaging measurement parameters had high intra-observer and inter-observer reliability (Table 2). The TT-TG distance was significantly reduced at follow-up compared with that before surgery (27.91 ± 1.95 mm vs 12.33 ± 1.07 mm, *P* < 0.001). The CSA of VMO at 5 mm below the upper pole of the patella was significantly increased at follow-up compared with that before surgery (473.06 ± 106.32 mm^2^ vs 562.97 ± 157.90 mm^2^, *P* < 0.001). The CSA of VMO at the upper pole of the patella was significantly increased at follow-up compared with that before surgery (641.23 ± 188.45 mm^2^ vs 700.23 ± 177.55 mm^2^, *P* = 0.029). The CSA of VMO at 5 mm above the upper pole of the patella was significantly increased at follow-up compared with that before surgery (788.25 ± 238.62 mm^2^ vs 849.79 ± 180.84 mm^2^, *P* = 0.018). The CSA/BW at 5 mm below the upper pole of the patella was significantly increased at follow-up compared with that before surgery (7.83 ± 2.52 mm^2^/kg vs 9.22 ± 3.54 mm^2^/kg, *P* < 0.001). The CSA/BW at the upper pole of the patella was significantly increased at follow-up compared with that before surgery (10.48 ± 3.62 mm^2^/kg vs 11.42 ± 4.14 mm^2^/kg, *P* = 0.020). The CSA/BW at 5 mm above the upper pole of the patella was significantly increased at follow-up compared with that before surgery (12.86 ± 4.65 mm^2^/kg vs 13.68 ± 3.86 mm^2^/kg, *P* = 0.017) (Table 3).

**Table 2:** Intra-observer and inter-observer reliability of imaging measurement parameters

Imaging measurement parameters	Intra-observer ICC (95% CI)	Inter-observer ICC (95% CI)
Before surgery		
CSA of VMO (− 5 mm)	0.993 (0.983–0.997)	0.968 (0.926–0.986)
CSA of VMO (0 mm)	0.994 (0.986–0.997)	0.979 (0.951–0.991)
CSA of VMO (+ 5 mm)	0.994 (0.985–0.997)	0.982 (0.959–0.992)
TT-TG distance	0.992 (0.981–0.997)	0.986 (0.966–0.994)
At follow-up		
CSA of VMO (− 5 mm)	0.995 (0.988–0.998)	0.990 (0.976–0.996)
CSA of VMO (0 mm)	0.997 (0.993–0.999)	0.987 (0.970–0.995)
CSA of VMO (+ 5 mm)	0.995 (0.988–0.998)	0.972 (0.935–0.988)
TT-TG distance	0.993 (0.984–0.997)	0.979 (0.951–0.991)

*ICC* intraclass correlation coefficient, *CI* confidence interval, *CSA of VMO(− 5 mm)* the cross-sectional area of vastus medialis oblique at 5 mm below the upper pole of the patella, *CSA of VMO(0 mm)* the cross-sectional area of vastus medialis oblique at the upper pole of the patella, *CSA of VMO(*+ *5 mm)* the cross-sectional area of vastus medialis oblique at 5 mm above the upper pole of the patella, *TT-TG* tibial tubercle-trochlear groove

**Table 3:** Comparison of measurement parameters before surgery and at follow-up

Parameters	Before surgery	At follow-up	*P* value
TT-TG distance	27.91 ± 1.95 mm	12.33 ± 1.07 mm	< 0.001
CSA of VMO (− 5 mm)	473.06 ± 106.32 mm^2^	562.97 ± 157.90 mm^2^	< 0.001
CSA of VMO (0 mm)	641.23 ± 188.45 mm^2^	700.23 ± 177.55 mm^2^	0.029
CSA of VMO (+ 5 mm)	788.25 ± 238.62 mm^2^	849.79 ± 180.84 mm^2^	0.018
CSA/BW (− 5 mm)	7.83 ± 2.52 mm^2^/kg	9.22 ± 3.54 mm^2^/kg	< 0.001
CSA/BW (0 mm)	10.48 ± 3.62 mm^2^/kg	11.42 ± 4.14 mm^2^/kg	0.020
CSA/BW (+ 5 mm)	12.86 ± 4.65 mm^2^/kg	13.68 ± 3.86 mm^2^/kg	0.017

*TT-TG* tibial tubercle-trochlear groove, *CSA of VMO(− 5 mm)* the cross-sectional area of vastus medialis oblique at 5 mm below the upper pole of the patella, *CSA of VMO(0 mm)* the cross-sectional area of vastus medialis oblique at the upper pole of the patella, *CSA of VMO(*+ *5 mm)* the cross-sectional area of vastus medialis oblique at 5 mm above the upper pole of the patella, *CSA/BW (− 5 mm)* the ratio of cross-sectional area of vastus medialis oblique to body weight at 5 mm below the upper pole of the patella, *CSA/BW (0 mm)* the ratio of cross-sectional area of vastus medialis oblique to body weight at the upper pole of the patella, *CSA/BW (*+ *5 mm)* the ratio of cross-sectional area of vastus medialis oblique to body weight at 5 mm above the upper pole of the patella

## Discussion

The main findings of this study supported the theory that tibial tubercle transfer combined with MPFL reconstruction was beneficial in restoring CSA of VMO in patients with RPD.

Research showed that MPFL was very vulnerable to injury in lateral patella dislocation [5]. At the same time, it was stated that the MPFL played an important role in maintaining the stability of patella [31]. Studies had shown that MPFL reconstruction was beneficial to restore the function of the MPFL, strengthen the stability of the patella, and reduce the occurrence of dislocation [32, 33]. In clinical work, many researchers agreed that TT-TG distance less than 20 was appropriate [34]. Researchers claimed that the increase in TT-TG distance would aggravate the lateral stress of patella in patients and even lead to the recurrence of patellar dislocation [35]. Tibial tubercle transfer reduced TT-TG distance by moving the tibial tubercle inward in response to the lateralization of the tibial tubercle relative to the femoral trochlear groove [36]. The reduction of TT-TG distance to a relatively suitable range could enhance the stability of patella and reduce the occurrence of dislocation [37, 38]. There were many studies on tibial tubercle transfer for the treatment of RPD [32, 39, 40]. Researchers had found that when used properly, additional tibial tubercle transfer was highly beneficial [13]. However, part of researchers thought that the additional assistance provided by tibial tubercle transfer was insufficient to compensate for the additional complications and risks associated with the operation [41]. Therefore, the appropriate surgical method for the treatment of RPD is still controversial. In this study, tibial tubercle transfer combined with MPFL reconstruction reduced the recurrence of patellar dislocation while reducing TT-TG distance.

It had been shown that patients with RPD had atrophy of the VMO and reduced CSA [17]. At present, there are many methods to measure muscle, such as tape measure, ultrasound, CT, and MRI [42–46]. We chose CT as the measurement method to not only measure the CSA of VMO, but also accurately identify the corresponding bone markers [29, 45]. As for the measured position, studies had shown that the VMO mainly existed in the distal vastus medial [27]. Researchers used the proximal end of the patella and its adjacent upper and lower layers as the sites to measure the CSA of VMO [28]. This parameter had also been measured in the maximum transverse diameter of the patella and the patella layers above it [29]. Based on previous studies, we measured CSA of VMO at 5 mm below the upper pole of the patella, the upper pole of the patella, and 5 mm above the upper pole of the patella, in order to compare the change of the CSA of VMO of the same part before surgery and at follow-up. Studies had confirmed that the CSA of muscles was related to the maximum contractility of muscles [47]. The VMO was a very important dynamic stabilizing device for the inner side of the patella. Atrophy of VMO would affect the stability of the patella during movement [17, 48]. Therefore, whether the reduction of CSA of VMO can be alleviated had become the focus of our observation and research. In this study, the CSA of VMO increased at 5 mm below the upper pole of the patella, the upper pole of the patella, and 5 mm above the upper pole of the patella in patients undergoing surgery, indicating that the atrophy of the VMO was alleviated after treatment, which was of great significance for maintaining the stability of the patella and preventing the recurrence of patella dislocation. In addition, although we included all RPD patients with epiphyseal closure, their BW may have changed during the follow-up period, although no statistically significant difference was observed in this study. Research showed that age, weight may have a certain impact on the human muscle volume [49]. There were studies using ratio of CSA of muscle to BW as an indicator to reduce the impact of weight change during follow-up [30, 49]. The CSA/BW also increased at 5 mm below the upper pole of the patella, the upper pole of the patella, and 5 mm above the upper pole of the patella, showing that the atrophy of VMO was alleviated after treatment after reducing the effects of weight. For the recovery of the muscle strength of patients with patella dislocation, researchers suggested scientific rehabilitation exercise [50–52]. Researchers had reported that quadriceps functional exercises can improve patellar stability and reduce pain in patients [53–55]. Some researchers thought it was necessary to strengthen the VMO [50].

In conclusion, after tibial tubercle transfer combined with MPFL reconstruction, CSA of VMO increased in patients with RPD, which will help to enhance patellar stability and reduce recurrence.

There were several limitations to this study. First of all, CT was not conducive to the observation of quadriceps, which may affect the observation of the real quadriceps. Secondly, our study design was retrospective and the number of patients included was insufficient. Thirdly, the procedure varied slightly from patient to patient, although it all depended on the patient's situation. Fourthly, patients had slightly different anatomical risk factors for patellofemoral instability, which, although not the focus of this study, may affect the course of postoperative recovery. Fifthly, only a small amount of clinical data was collected, so it remained unclear what relevance these results had at all. Another limitation of the study was that there was no control group. These factors may influence the results of the study.

## Data Availability

All of the data and materials are available.

## Abbreviations

RPD: Recurrent patellar dislocation
MPFL: Medial patellofemoral ligament
VMO: Vastus medialis oblique
TT-TG: Tibial tubercle-trochlear groove
CSA: Cross-sectional area
BW: Body weight
BMI: Body mass index
CDI: Caton–Deschamps index
PACS: Picture archiving and communication system
CSA/BW: The ratio of cross-sectional area of vastus medialis oblique to body weight
ICC: Intraclass correlation coefficients
